# Genetic Disruption of 21-Hydroxylase in Zebrafish Causes Interrenal Hyperplasia

**DOI:** 10.1210/en.2017-00549

**Published:** 2017-09-13

**Authors:** Helen Eachus, Andreas Zaucker, James A. Oakes, Aliesha Griffin, Meltem Weger, Tülay Güran, Angela Taylor, Abigail Harris, Andy Greenfield, Jonathan L. Quanson, Karl-Heinz Storbeck, Vincent T. Cunliffe, Ferenc Müller, Nils Krone

**Affiliations:** 1 Department of Biomedical Science, The Bateson Centre, Sheffield S10 2TN, United Kingdom; 2 Centre for Endocrinology, Diabetes, and Metabolism, College of Medical and Dental Sciences, University of Birmingham, Birmingham B15 2TT, United Kingdom; 3 Academic Unit of Child Health, Department of Oncology and Metabolism, University of Sheffield, Sheffield S10 2TG, United Kingdom; 4 Mammalian Genetics Unit, Medical Research Council, Harwell Institute, Oxfordshire OX11 0RD, United Kingdom; 5 Department of Biochemistry, Stellenbosch University, Stellenbosch, Matieland 7602, South Africa; 6 Institute of Cancer and Genomic Sciences, University of Birmingham, College of Medical and Dental Sciences, Birmingham B15 2TT, United Kingdom

## Abstract

Congenital adrenal hyperplasia is a group of common inherited disorders leading to glucocorticoid deficiency. Most cases are caused by 21-hydroxylase deficiency (21OHD). The systemic consequences of imbalanced steroid hormone biosynthesis due to severe 21OHD remains poorly understood. Therefore, we developed a zebrafish model for 21OHD, which focuses on the impairment of glucocorticoid biosynthesis. A single 21-hydroxylase gene (*cyp21a2*) is annotated in the zebrafish genome based on sequence homology. Our *in silico* analysis of the 21-hydroxylase (Cyp21a2) protein sequence suggests a sufficient degree of similarity for the usage of zebrafish *cyp21a2* to model aspects of human 21OHD *in vivo*. We determined the spatiotemporal expression patterns of *cyp21a2* by whole-mount *in situ* hybridization and reverse transcription polymerase chain reaction throughout early development. Early *cyp21a2* expression is restricted to the interrenal gland (zebrafish adrenal counterpart) and the brain. To further explore the *in vivo* consequences of 21OHD we created several *cyp21a2* null-allele zebrafish lines by using a transcription activator–like effector nuclease genomic engineering strategy. Homozygous mutant zebrafish larvae showed an upregulation of the hypothalamic–pituitary–interrenal (HPI) axis and interrenal hyperplasia. Furthermore, Cyp21a2-deficient larvae had a typical steroid profile, with reduced concentrations of cortisol and increased concentrations of 17-hydroxyprogesterone and 21-deoxycortisol. Affected larvae showed an upregulation of the HPI axis and interrenal hyperplasia. Downregulation of the glucocorticoid-responsive genes *pck1* and *fkbp5* indicated systemic glucocorticoid deficiency. Our work demonstrates the crucial role of Cyp21a2 in glucocorticoid biosynthesis in zebrafish larvae and establishes an *in vivo* model allowing studies of systemic consequences of altered steroid hormone synthesis.

## Teaser

Zebrafish larvae with disrupted 21-hydroxylase have interrenal hyperplasia and systemic glucocorticoid deficiency.

Steroid hormones are key regulators of sex development, behavior, body homeostasis, and metabolism. Deficiencies of steroid hormone synthesis and action are common causes of disorders of sex development including congenital adrenal hyperplasia (CAH). CAH ranks among the most common inherited metabolic endocrine disorders, occurring in ~1 in 10,000 to 1 in 15,000 affected individuals (1, 2). It is associated with morbidity and mortality (3, 4) and represents a classic example of conditions with severe systemic consequences due to altered steroid hormone synthesis. The majority of CAH cases are caused by 21-hydroxylase (CYP21A2) deficiency (21OHD) resulting from inactivating mutations in the *CYP21A2* gene. CYP21A2 is a cytochrome P450 enzyme located in the endoplasmic reticulum, which in humans catalyzes the conversion of 17-hydroxyprogesterone (17OHP) to 11-deoxycortisol, a cortisol precursor, and the conversion of progesterone to 11-deoxycorticosterone, a precursor of aldosterone in humans (2). Disruption of this pathway renders patients unable to synthesize cortisol efficiently and results in the overproduction of adrenocorticotropic hormone (ACTH) by the pituitary because of diminished negative feedback. The stimulation of the adrenal cortex by ACTH in turn leads to overproduction of cortisol precursors, which are diverted to the biosynthesis of sex hormones, leading to sex hormone excess.

The major challenge in the field of steroid endocrinology is a substantial lack of understanding of systemic consequences of genetically disrupted steroid hormone synthesis causing CAH. Increasing evidence suggests that steroid hormone precursors altered in inborn errors of steroidogenesis, such as 21OHD, can alter glucocorticoid action (5). However, the homeostatic consequences on the whole organism remain elusive. Although a murine model of 21OHD due to naturally occurring mutations (6) has led to insights of adrenal development (7, 8), only limited insights regarding systemic effects of altered steroid synthesis on the whole organism have been gained because of difficulties maintaining these mice.

Zebrafish are a comprehensive *in vivo* model organism for studying adrenal steroid hormone biosynthesis (9–11). Importantly, zebrafish share extensive homologies with humans in terms of their genome and the structure and function of several neural and physiological systems, including the neuroendocrine axis (12). In contrast to mice, zebrafish generate cortisol as the main glucocorticoid, with the same intermediates as humans, and as diurnal animals they follow a similar circadian rhythm (13). Thus, we anticipate zebrafish to represent a highly suitable model organism to explore the systemic consequences of genetically altered steroid hormone biosynthesis.

Only very limited information is available on zebrafish 21-hydroxylase (Cyp21a2) despite the well-recognized biosynthesis of cortisol in zebrafish (14), for which a 21-hydroxylation step is crucial. Therefore, we have developed a Cyp21a2-deficient zebrafish model by using a transcription activator–like effector nuclease (TALEN) strategy to define the role of zebrafish Cyp21a2. Cyp21a2-deficient zebrafish have a number of systemic hallmark features of human 21OHD, including upregulation of the hypothalamic–pituitary–interrenal (HPI) axis, interrenal hyperplasia, pathognomonic steroid hormone profiles, and reduced systemic glucocorticoid-mediated expression of target genes. Thus, we believe that this model will not only define crucial steps of the steroidogenic pathway in zebrafish but also serve as a model to delineate systemic effects of glucocorticoid deficiency specific to 21OHD.

## Materials and Methods

### Zebrafish husbandry

Zebrafish were maintained in a recirculating system (ZebTECTM, Tecniplast®, Kettering, UK, and Sheffield, UK) at 28.5°C in a 10:14 dark/light photoperiod. Embryos were obtained by natural spawning and incubated at 28.5°C in E3 medium (5 mmol/L NaCl, 0.17 mmol/L KCl, 0.33 mmol/L CaCl_2_, 0.33 mmol/L MgSO_4_) containing 2 μg/mL gentamycin. The developmental stages were determined according to hours postfertilization (hpf), and morphological features were as previously described (15). All procedures were approved by the Home Office, United Kingdom and carried out in line with the Animals (Scientific Procedures) Act 1986.

### Comparison of vertebrate CYP21A2 protein sequences

CYP21A2 protein sequences were retrieved from the National Center for Biotechnology Information: NP_000491.4, *Homo sapiens,* human; XP_003311237.1, *Pan troglodytes,* chimpanzee; NP_001181556.1, *Macaca mulatta,* macaque; NP_001003335.1, *Canis lupus familiaris,* dog; NP_001013614.1, *Bos taurus,* cattle; NP_034125.2, *Mus musculus,* mouse; NP_476442.2, *Rattus norvegicus,* rat; NP_001092828.1, *Gallus,* chicken; XP_002941314.2, *Xenopus (Silurana) tropicalis,* frog; and XP_009290467.1, (*Danio rerio,* zebrafish. Multiple sequence alignments were carried out *via* the online tool Clustal Omega (www.ebi.ac.uk/Tools/msa/clustalo) (16, 17) with the default settings.

### Gene expression analysis by reverse transcription polymerase chain reaction

Total RNA was extracted and complementary DNA (cDNA) synthesized as previously described (18). We used 100 ng of cDNA as template in 20-µL polymerase chain reaction (PCR) reactions set up in MegaBlue Master Mix (Microzone, Haywards Heath, United Kingdom), including 300 nM for each *cyp21a2* primer. The cycler program consisted of an initial activation at 94°C for 2 minutes followed by 36 cycles of 94°C for 30 seconds, 60°C for 30 seconds, and 72°C for 30 seconds, before a final elongation for 10 minutes at 72°C. The whole PCR was loaded for analysis on a 2% agarose gel. No-template controls and a PCR with a *cyp21a2* coding sequence containing pGEM-T Easy Vector (Promega, Southampton, United Kingdom) served as negative and positive controls, respectively.

### Whole-mount RNA *in situ* hybridization

Whole-mount RNA *in situ* hybridization (WISH) was carried out according to a standard protocol as previously described (19). For the generation of *cyp21a2* probes, a pGEM-T Easy Vector (Promega) containing the *cyp21a2* cDNA (ENSDART00000150512) was cut with *Nde*I and *Sac*II restriction enzymes to generate templates for *in vitro* transcription. We then synthesized digoxigenin-labeled *cyp21a2* sense and antisense probes through *in vitro* transcription of 1 µg template with T7 (*Nde*I, sense) and SP6 (*Sac*II, antisense) polymerases by using the reagents of the DIG RNA Labeling Kit (11175025910; Roche, Burgess Hill, United Kingdom).

For generation of the *cyp17a2* probe, a 996-bp fragment of the *cyp17a2* transcript (NM_001105670.1) was amplified from 3 days postfertilization (dpf) AB wild-type cDNA using the primer pair forward: GGCTGACAGTCTGTGTGAGG and reverse: GTGTAGCGCTCAGGCTGTAA. The PCR product was cloned into pGEM-T Easy Vector, and the insert was sequenced. The *cyp17a2* probe was generated with T7/*Nde*I for sense and SP6/*Nco*I for the antisense probe. The *pomca* probe was generated as previously described (20).

### Generation of *cyp21a2* mutants by TALENs

The pair of TALENs targeting exon 2 of the *cyp21a2* gene was generated with the Golden Gate TALEN Kit (Addgene, Cambridge, MA). TALEN target sites were determined by TAL Effector Nucleotide Targeter Version 2.0 (21). The sequences of the repeat-variable di-residues (RVDs) in the TAL Effector DNA-binding domains were RVD TALE1 (left): NG-HD-NG-NH-NH-NG-HD-HD-NG-HD-NH-HD-NG-HD-NG-HD and RVD TALE2 (right): NG-NH-NH-NH-HD-NH-NI-NH-NI-NG-HD-HD-NI-NH-HD-NI-NG-NH-NG. TALEN mRNA was synthesized with an SP6 polymerase mMessage mMachine Kit (Life Technologies, Waltham, MA) after 1 µg of plasmid DNA was digested with *Not*I.

TALENs were injected into one-cell-stage embryos, with 1 nL of injection solution containing 50 or 150 ng/µL of each TALEN diluted in nuclease-free water (Promega) plus 0.1% phenol red. F0 generations were grown to adulthood from the injected embryos and screened for transmission of *cyp21a2* mutations. Identified founders were outcrossed to fish to generate F1 generations. The F1 generation fish were screened for heterozygous *cyp21a2* mutations. F1 fish with defined *cyp21a2* mutant alleles were outcrossed to their respective genetic backgrounds to generate the F2 generation. The heterozygous mutant fish of the F2 generation were incrossed to study *cyp21a2* mutant phenotypes in embryos and larvae.

### Genotyping *cyp21a2* mutants

Genomic DNA was extracted from fin clips or whole larvae. For the extraction of genomic DNA, samples were lysed in 20 µL (embryos) to 40 µL (fin clips) of rapid PCR buffer. PCR amplification of *cyp21a2* exon 2 was carried out in 20-µL PCRs with 300 nM of each primer (forward: CTCTCGTGGGCTAAACAAGC and reverse: ACATGTATCCACCATTTGCG) and 1 µL genomic DNA template in MegaMix-Blue (Microzone). The PCR program consisted of an initial activation at 94°C for 2 minutes followed by 36 cycles of 94°C for 30 seconds, 58°C for 30 seconds, and 72°C for 30 seconds, before a final elongation for 10 minutes at 72°C. Ten µL PCR product was then digested with BseYI in a 30-µL reaction. The digests were analyzed on a 1% agarose gel. The 179-bp product is cleaved in wild-type samples only, giving 103-bp and 76-bp products (Supplemental Fig. 1).

### Analysis of visual background adaptation

Zebrafish larvae can adjust their pigmentation to match their surrounding environments, a form of crypsis. To identify *cyp21a2* mutants, larvae were sorted according to visual background adaptation (VBA) response at 96 hpf. The assay was carried out as previously described (18).

### Area measurements on images from WISH

Larvae derived from incrosses of adult *cyp21a2^UOB2122/+^* and *cyp21a2^UOB2123/+^* fish were raised to 120 hpf, at a density of ~100 larvae per petri dish (20 mL). Ten VBA+ larvae and 10 VBA− siblings were fixed and processed through *cyp17a2* WISH together in one 1.5-mL tube. Images depicting a dorsal view of the interrenal gland for individual larvae after WISH were cropped and arranged in Adobe Photoshop. ImageJ software was used to set a color threshold to distinguish background from the dark purple WISH staining. The number of stained pixels was then quantified in ImageJ.

### Induction of osmotic stress

Sodium chloride treatments of 250 mM (in E3) were given at 120 hpf for 20 minutes.

### Steroid hormone measurements

After the washing of chemical treatments, each clutch of 150 larvae was transferred into a silanized test tube and snap frozen on dry ice. We added 1 mL of phosphate-buffered saline to the sample, and the cells were lysed via four rounds of freeze thawing. After lysis the samples were homogenized with a pestle homogenizer. We added 20 µL of a solution containing a mix of deuterated steroids in MeOH to the samples to provide an internal reference for normalization. A calibration series of a mix of steroids was generated in 50% MeOH. Steroids were extracted from the samples with 3 mL methyl *tert*-butyl ether (MTBE). The upper MTBE phase with the extracted steroids was transferred into clean glass test tubes. The extraction step was repeated with an additional 3 mL MTBE. The upper MTBE phase was added to the extract from the first round of extraction, and the pooled solvent evaporated under a stream of nitrogen. The dried steroids were subsequently resuspended in 150 μL 50% methanol and were separated and quantified with an Acquity UPLC System (Waters, Milford, CT) coupled to a Xevo TQ-S tandem mass spectrometer (Waters). Chromatographic separation was achieved with an ultra-performance liquid chromatography high-strength silica T3 column (2.1 mm × 50 mm, 1.8 μm) (Waters) as previously described (22).

### Comparative gene expression analysis by quantitative PCR

At 120 hpf, clutches of 30 VBA+ and 30 VBA− larvae were snap frozen in liquid nitrogen. Total RNA was extracted and reverse transcribed as described earlier. Quantitative polymerase chain reaction (qPCR) methods and primers sequences for *pomca,* *fkbp5,* and *pck1* have been previously described (18). Expression levels for each gene were normalized to *gapdh,* and fold change values were generated relative to wild-type control levels. Biological replicates were standardized as previously described (23).

### Optical projection tomography

Samples were embedded in 1% agarose in water, dehydrated for 24 hours in 100% MeOH, and then cleared with 1:2 benzyl alcohol/benzyl benzoate for 48 hours. Samples were imaged with a bespoke optical projection tomography (OPT) scanner (24) with white light, followed by ultraviolet light. Scans were reconstructed with NRecon (Bruker microCT) and further processed in Fiji to generate three-dimensional images.

### Statistical analysis

Statistical analyses and graphics were prepared in R version 3.3.0. For the comparison of means between two samples, unpaired *t* tests were used to test for significant differences. For the comparison of means of more than two samples, one- or two-way analysis of variance (ANOVA) was used, followed by Tukey *post hoc* test, when a significant interaction was detected. Fold change values for *pomca* expression measured via qPCR were log transformed before analysis via two-way ANOVA, to meet the assumptions of the test (untransformed data are plotted).

## Results

### Zebrafish 21-hydroxylase (*cyp21a2*) single-copy gene

A single copy of the 21-hydroxylase gene, *cyp21a2,* was identified in the zebrafish. The *cyp21a2* gene resides on chromosome 16 and has two predicted protein-coding splice variants, of 533 and 523 amino acids, each with 12 exons. Zebrafish Cyp21a2 shares homology with other vertebrate CYP21 proteins. To determine the evolutionary conservation of the zebrafish Cyp21a2, protein sequence analysis was performed *in silico*. Zebrafish Cyp21a2 shares high sequence homology with other teleost fish and high sequence homology with the human 21-hydroxylase protein (40.71%) (Supplemental Table 1).

### Temporospatial expression of the *cyp21a2* gene

**Figure 1.**
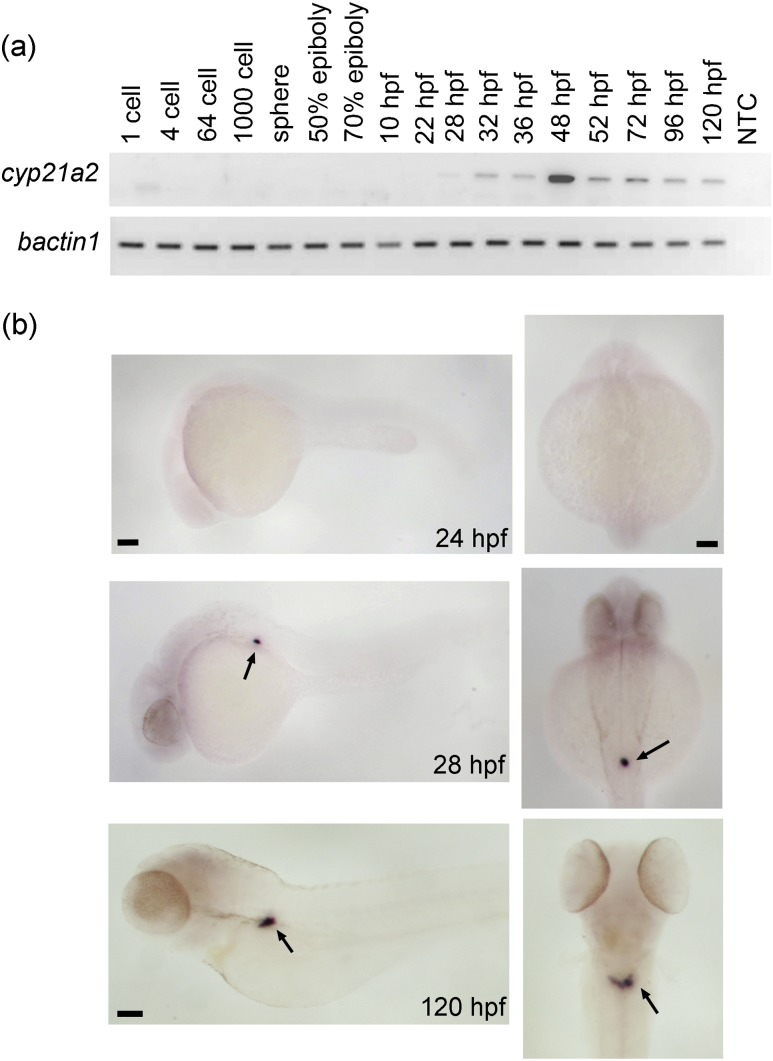
Zebrafish *cyp21a2* expression is largely restricted to the interrenal gland. (a) Analysis of *cyp21a2* expression during embryonic and early larval development by RT-PCR with *b-actin1* as an internal standard (gel images inversed). A no-template control sample (NTC) served as negative control. The onset of detectable *cyp21a2* expression is at 28 hpf, after which *cyp21a2* continues to be expressed at all stages examined. (b) Images of wild-type larvae at different stages after WISH against *cyp21a2*. Left panels show lateral view, with head to the left; right panels show a dorsal view. No staining is seen in 24-hpf embryos. In 28-hpf embryos and 120-hpf larvae, strong staining is observed in the interrenal gland region, with some faint signal detected at 120 hpf in the head (arrows). Scale bar: 0.1 mm.

Expression of the *cyp21a2* gene was analyzed by reverse transcription polymerase chain reaction (RT-PCR) during early zebrafish development. The expression of *cyp21a2* started around the late segmentation period (28 hpf) and was maintained to 120 hpf [Fig. 1(a)]. WISH carried out at 24, 28, and 120 hpf showed that *cyp21a2* has high expression within the interrenal gland from 28 hpf [Fig. 1(b)].

### Generation of *cyp21a2* null alleles in zebrafish with TALENs

To further study the function of 21-hydroxylase in zebrafish, we disrupted the *cyp21a2* gene by using a TALEN strategy. The TALEN binding sites were chosen within exon 2 to generate an early 5-prime disruption into the *cyp21a2* gene. The left TALEN targeted 17 nucleotides of the *cyp21a2* gene, and the right TALEN targeted 20 nucleotides. Each was separated by a spacer sequence of 15 bp (Supplemental Fig. 1A). The genomic disruption in injected embryos was confirmed by a BseYI restriction digest after PCR. Subsequently, three different heritable alleles were identified within the targeted spacer region, of which two lines were maintained (*cyp21a2^uob2122^* and *cyp21a2^uob2123^*). The *cyp21a2^uob2122^* mutant line has a 14-bp deletion (c.del211–224), leading to a frameshift with a premature stop at amino acid 96 (p.P70 *fs*26X). The second line carried a deletion of 13 nucleotides (c.del212–224), causing a frameshift and an early stop codon at position 83 (p.P70*fs*13X). Thus, both mutations are predicted to result in abolished 21-hydroxylase function. Genotyping of wild-type, heterozygous, and homozygous mutant larvae was performed by BseYI restriction digest after PCR (Supplemental Fig. 1B).

### Impaired VBA response and interrenal hyperplasia in *cyp21a2* mutant larvae

The homozygous *cyp21a2* mutants were characterized during the first 5 days of zebrafish development to determine the requirement of *cyp21a2* for steroid hormone biosynthesis in developing zebrafish larvae. *cyp21a2* homozygous mutants were morphologically similar to control siblings during this time (Supplemental Fig. 2A). Because background adaptation in zebrafish has been associated with impaired glucocorticoid synthesis (18) and action (25), we subjected larvae to VBA analysis.

Larvae from a *cyp21a2* heterozygous incross were analyzed by VBA assessment at 120 hpf and sorted into dark pigmentation and light pigmentation (Supplemental Fig. 2A). Genotyping of 96 larvae (*cyp21a2^UOB2122^* and *cyp21a2^UOB2123^*) with impaired VBA revealed that most larvae were homozygous for the *cyp21a2* allele (Supplemental Fig. 2B). Larvae capable of light adaptation were always either *cyp21a2* wild type (*cyp21a2^+/+^*) or *cyp21a2* heterozygotes (*cyp21a2^+/−^*). Only a small percentage of heterozygous *cyp21a2 ^UOB2122/+^* showed an altered VBA response, confirming that *cyp21a2* homozygous mutants can be reliably distinguished from their siblings by VBA assessment.

**Figure 2.**
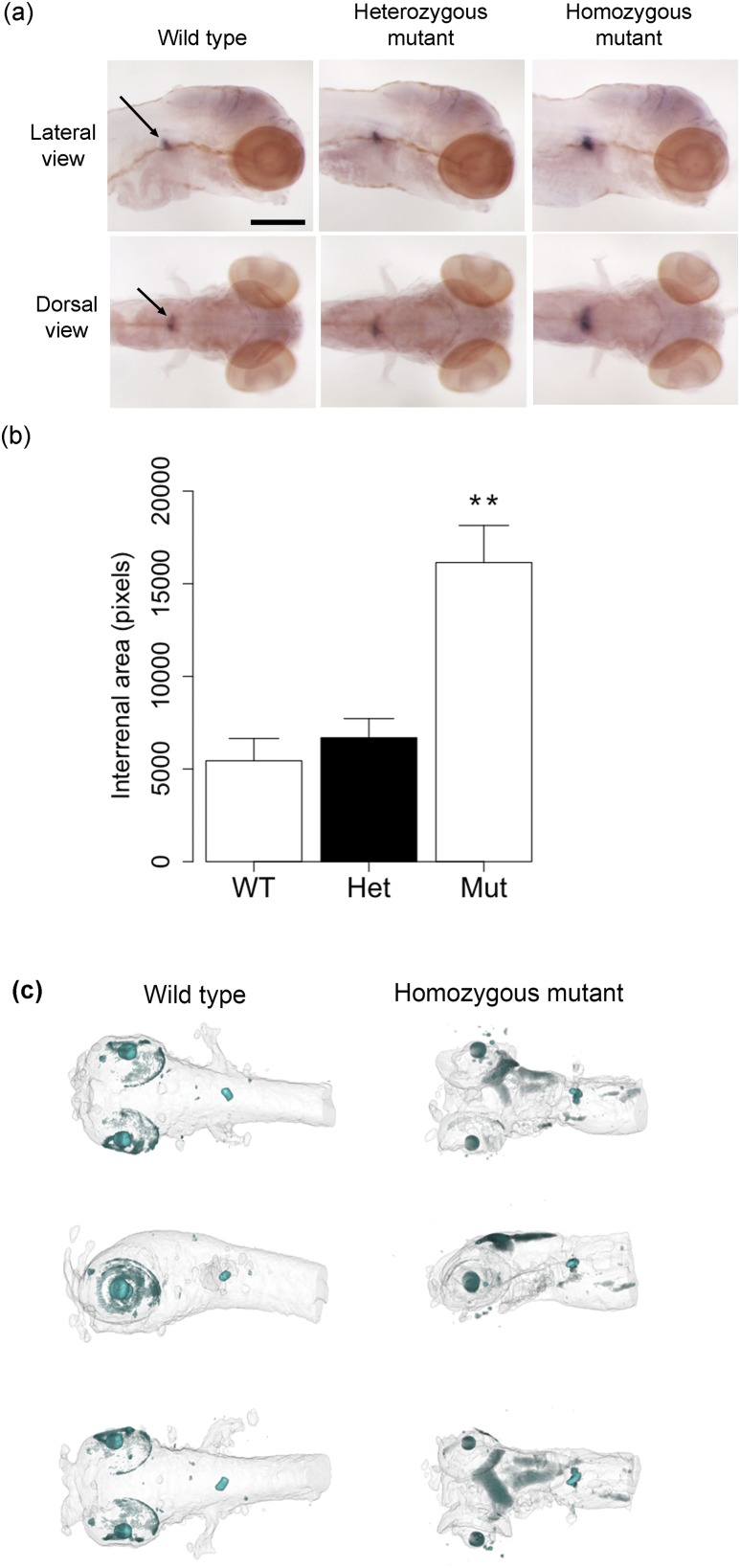
Zebrafish *cyp21a2* mutants have enlarged interrenal tissue at 120 hpf. (a) Expression of *cyp17a2* in 120-hpf *cyp21a2^uob2122^* wild-type, heterozygous, and homozygous *cyp21a2^uob2122^* mutant larvae in lateral (upper panel) and dorsal (lower panel) views. The area of *cyp17a2*-positive interrenal tissue (arrows) is enlarged in homozygous mutants. n = 6 each. Scale bar: 0.25 mm. (b) Quantification of the area of *cyp17a2*-positive interrenal tissue in 120-hpf *cyp21a2^uob2122^* larvae. The interrenal tissue is significantly larger in homozygous mutants (Mut) than in wild-type (WT) and heterozygous (Het) siblings (ANOVA: *F* = 12.15, df = 2,15, *P* = 0.0007; Tukey: WT *vs*. Het *P* = 0.895, WT *vs*. Mut *P* = 0.003, Het *vs*. Mut *P* = 0.003). **P < 0.01 compared with wild types and heterozygotes. n = 4 to 8 each. (c) OPT imaging of 120-hpf *cyp21a2* larvae after WISH for *cyp17a2* reveals an enlarged interrenal gland in *cyp21a2^uob2122^* homozygous mutants (right) compared with wild-type siblings (left). Whole-mount ventral (upper), lateral (middle), and dorsal (lower) views are shown.

At 120 hpf, *cyp21a2^UOB2122^* homozygous larvae already showed an increased staining in the WISH analysis with an interrenal specific *cyp17a2* probe [Fig. 2(a)]. A quantitative analysis confirmed that interrenal size was significantly greater in homozygous mutants than in wild types and heterozygotes [Fig. 2(b)]. Interrenal hyperplasia was confirmed in the *cyp21a2^UOB2122^* homozygous mutant larvae at 5 dpf using a three-dimensional reconstruction of OPT imaging of a WISH experiment with a *cyp17a2* probe [Fig. 2(c)]. This finding is probably caused by ACTH-induced interrenal hyperplasia presenting the correlate of congenital adrenal hyperplasia in humans.

### Impaired steroidogenesis in *cyp21a2* mutant zebrafish

**Figure 3.**
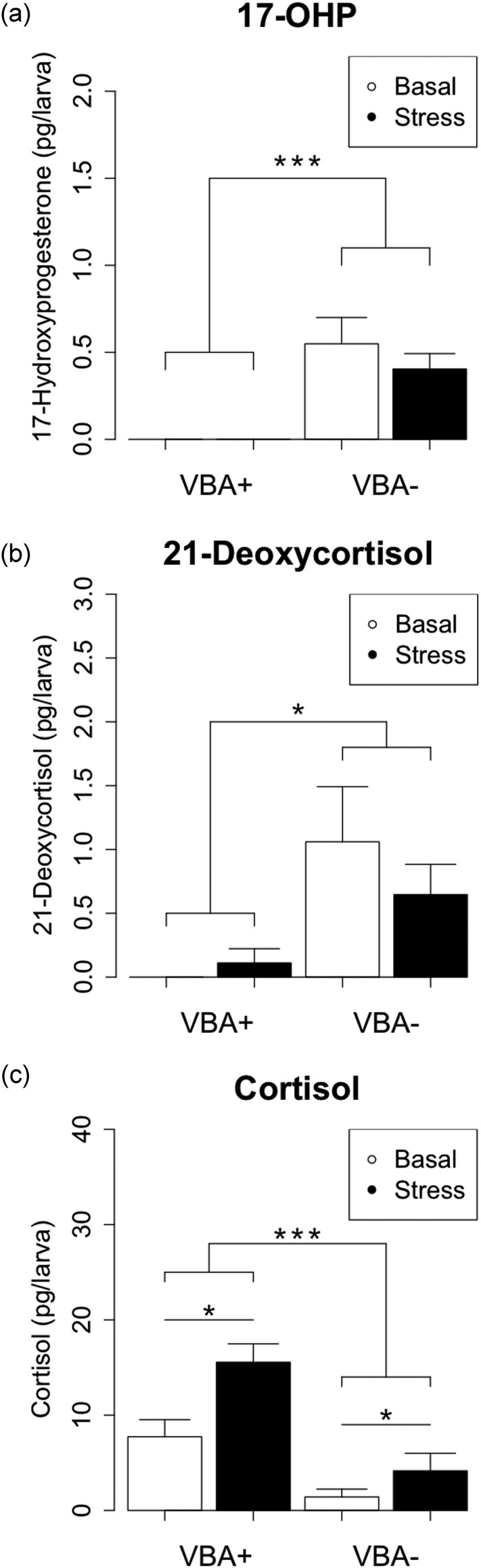
Zebrafish *cyp21a2* mutants have impaired steroidogenesis. Measurement of baseline and stress-induced concentrations of steroid hormones in 120-hpf *cyp21a2^uob2122^* VBA+ (wild-type) and VBA− (mutant) larvae by UPLC-MS/MS. (a) 17-OHP concentrations are significantly higher in VBA− (mutant) larvae than in VBA+ (wild-type) larvae, and levels are not significantly altered by stress treatment (two-way ANOVA: genotype *F* = 29.71, df = 1,8, ***P = 0.0006; stress *F* = 0.68, df = 1,8, *P* = 0.433; genotype/stress *F* = 0.68, df = 1,8, *P* = 0.433). n = 3 each. (b) 21-Deoxycortisol concentration is significantly higher in VBA− (mutant) larvae than in VBA+ (wild-type) larvae and is not significantly altered by stress treatment (two-way ANOVA: genotype *F* = 9.97, df = 1,8, *P = 0.013; stress *F* = 0.36, df = 1,8, *P* = 0.565; genotype/stress *F* = 1.08, df = 1,8, *P* = 0.329). n = 3 each. (c) Cortisol concentration is significantly lower in VBA− (mutant) larvae than in VBA+ (wild-type) larvae, and levels are significantly increased by stress treatment (ANOVA: genotype *F* = 28.63, df = 1,8, ***P = 0.0007; stress *F* = 10.19, df = 1,8, *P = 0.013; genotype/stress *F* = 2.35, df = 1,8, *P* = 0.163). n = 3 each.

At 4 dpf, *cyp21a2^UOB2122^* larvae were sorted into VBA+ and VBA− larvae (150 larvae each group), and samples were analyzed at 5 dpf. The steroid hormone profiling by ultra-performance liquid chromatography–tandem mass spectrometry (UPLC-MS/MS) revealed detection of 17OHP in VBA− larvae only, and this amount did not further increase with stress treatment [Fig. 3(a)]. Remarkably, 21-deoxycortisol, which is pathognomonic for 21OHD in humans, was detected at high concentrations only in VBA− larvae, biochemically proving 21OHD [Fig. 3(b)]. Concentrations of 21-deoxycortisol were not affected by stress treatment [Fig. 3(b)]. Additionally, UPLC-MS/MS revealed reduced capability of VBA− larvae to produce cortisol [Fig. 3(c)]. Both VBA+ and VBA− larvae exhibited an increase in cortisol concentrations in response to acute stress [Fig. 3(c)]. We were not able to detect mineralocorticoid precursors such as deoxycorticosterone and corticosterone by UPLC-MS/MS, nor did we detect androgen precursors such as androstenedione in VBA+ or VBA− zebrafish larvae (data not shown).

### Systemic changes due to cortisol deficiency

**Figure 4.**
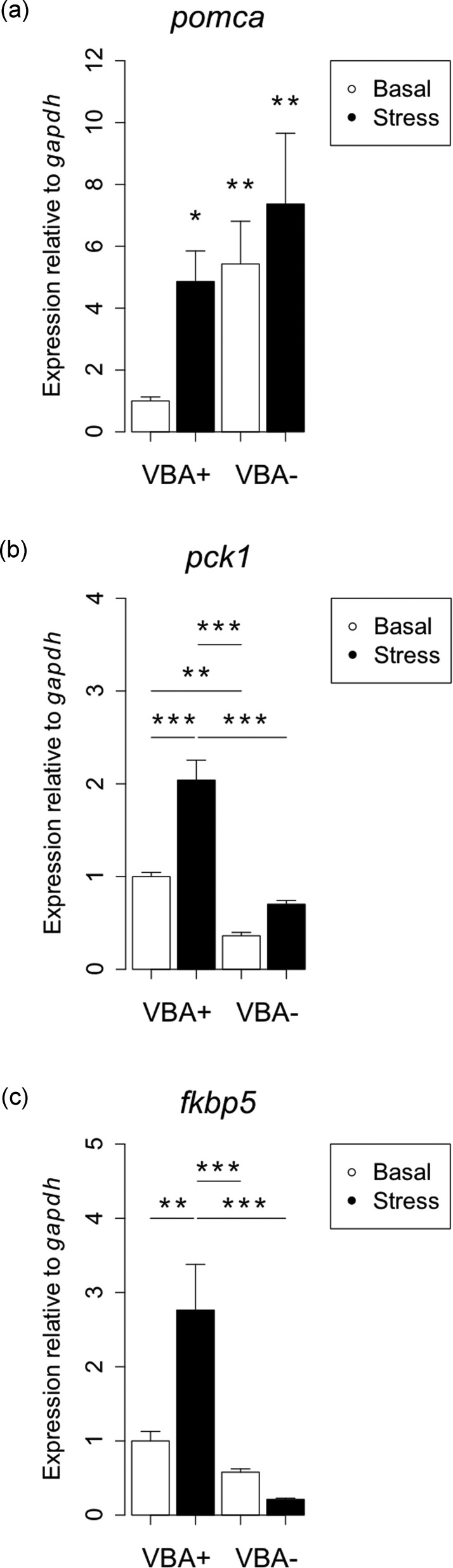
Zebrafish *cyp21a2* mutants have a dysregulated HPI axis. Analysis of baseline and stress-induced transcript levels of *pomca, fkbp5, pck1* in 120-hpf VBA+ (wild-type) *vs*. their VBA− (mutant) siblings by qPCR. Expression is relative to the control gene *gapdh*. (a) Expression of *pomca* is affected by VBA response (genotype) and stress treatment (two-way ANOVA: VBA/stress interaction *F* = 4.15, df = 1,20, *P* = 0.05). Expression increases in VBA+ larvae in response to stress (*P = 0.01), and expression in VBA− larvae under control (**P = 0.007) and stressed (**P = 0.002) conditions is higher than VBA+ baseline levels. Analysis carried out on log-transformed data; untransformed data are plotted. n = 6 each. (b) Expression of *pck1* is affected by VBA response and stress treatment (two-way ANOVA: VBA/stress interaction *F* = 9.59, df = 1,16, **P = 0.006). Expression is lower in VBA− larvae than in VBA+ larvae under baseline (***P = 0.005) and stressed (***P < 0.0001) conditions. Expression increases in VBA+ larvae in response to stress (***P < 0.0001), but stress has no effect on expression levels in VBA− larvae (*P* = 0.18). n = 5 each. (c) Expression of *fkbp5* is affected by VBA response and stress treatment (two-way ANOVA: VBA/stress interaction *F* = 11.38, df = 1,16, ***P = 0.003). Expression in VBA+ larvae increases in response to stress (**P = 0.005), but stress has no effect on VBA− expression levels (*P* = 0.84). Expression of *fkbp5* in VBA+ larvae under stress was significantly lower in VBA− than in VBA+ larvae (***P = 0.0001). n = 5 each.

The systemic effects of *cyp21a2* loss were analyzed by quantitative RT-PCR. In response to the impaired interrenal cortisol biosynthesis, significantly higher levels of *pomca* transcripts were observed in VBA− larvae when compared with VBA+ controls [Fig. 4(a)], indicating activation of the HPI axis. Whereas *pomca* transcript levels in VBA+ larvae increased in response to acute stress, no stress-induced increase was detected in VBA− larvae, where levels were as high as in stressed VBA+ larvae. These results were confirmed by WISH showing greater staining of pituitary tissue in *cyp21a2* homozygous mutants with a *pomca* probe compared with wild-type larvae (Supplemental Fig. 3).

In addition, the expression of the glucocorticoid-responsive gene *pck1* was significantly lower in VBA− larvae than in VBA+ controls [Fig. 4(b)]. Osmotic stress resulted in a significant increase of expression levels of *pck1* in VBA+ larvae but did not have a significant effect on *pck1* levels in VBA− larvae [Fig. 4(b)]. Similarly, osmotic stress did not increase expression levels of *fkbp5* in VBA− larvae, whereas VBA+ larvae showed significantly increased expression levels under stress [Fig. 4(c)]. Overall, this finding clearly demonstrates reduced transcriptional response to stress on global level of VBA− larvae when compared with VBA+ larvae (Fig. 4).

## Discussion

Zebrafish are increasingly used as model organisms in biomedical research, including the study of endocrine conditions, stress research, and the modeling of anxiety and depression (26–28). The hypothalamic–pituitary–adrenal axis in mammals and the HPI axis in fish play a crucial role in these scientific areas. However, the detailed biosynthetic pathway to cortisol in zebrafish remains partly elusive. Herein, we present clear *in vivo* evidence of the key role of 21-hydroxylase (Cyp21a2) in glucocorticoid biosynthesis in zebrafish larvae.

Some of the cytochrome P450 enzymes involved in steroid hormone biosynthesis such as P450 side chain cleavage (Cyp11a1 and Cyp11a2), 17-hydroxylase (Cyp17a1 and Cyp17a2), and P450 aromatase (Cyp19a1a and Cyp19a1b) remain duplicated and show a temporal-spatial or functional separation, whereas 3-beta-hydroxysteroid dehydrogenase (Hsd3b1) (29) and 11-hydroxylase (Cyp11c1) (30) exist as single-copy genes. Similar to the latter genes, zebrafish have a single copy of the 21-hydroxylase gene (*Cyp21a2*), indicating that the duplicated copy arising from the whole-genome duplication of the common teleost ancestor (31) has presumably been lost during evolution. An extensive database search did not reveal a second copy, and zebrafish *cyp21a2*, which shows an overall homology to the human *CYP21A2* ortholog of 41%, can therefore be assumed the functional gene in zebrafish.

### Disruption of *cyp21a2* leads to glucocorticoid deficiency

Expression of *cyp21a2* can be detected when the interrenal gland develops the ability of *de novo* cortisol synthesis (14). Expression of *cyp21a2* was localized mainly to the interrenal gland. By using TALENs to disrupt the open-reading frame of *cyp21a2,* we generated zebrafish mutant alleles with loss of the conserved functional domains of 21-hydroxylase. A mutant line harboring a 14-bp deletion was established and used to investigate the need of *cyp21a2* for cortisol biosynthesis.

VBA is observed from 96 hpf and is a rapid, reversible physiological process regulated by glucocorticoid receptor signaling in teleost fish (25). VBA involves the distribution or aggregation of melanin within the melanophore to blend into the surrounding environment (32). The VBA analysis of 5-dpf larvae subjected to lighter environments revealed that the overall majority of darker larvae correlated with zebrafish homozygous for the nonfunctional *cyp21a2^−/−^* genotype. In addition, homozygous *cyp21a2*^−/−^ showed an upregulation of the HPI axis, as indicated by increased expression levels of the *pomca* gene assessed by qPCR and *in situ* hybridization. This finding is similar to that in humans with primary adrenal insufficiency, who show hyperpigmentation due to upregulation of the hypothalamic–pituitary–adrenal axis with increased levels of POMC expression. Furthermore, this finding is consistent with observations in glucocorticoid-resistant (33) and glucocorticoid-deficient (18) zebrafish models, leading to a disruption of the negative HPI feedback loop.

### Cyp21a2 is a key enzyme in zebrafish cortisol biosynthesis

At 96 hpf, larvae from heterozygous incrosses were sorted according to their VBA response and collected at 120 hpf for steroid hormone analysis. VBA− larvae showed a pathognomonic steroid hormone profile with increased concentrations of 17OHP and the 21OHD marker steroid hormone 21-deoxycortisol resembling the hormonal constellation of glucocorticoid precursors observed in humans with 21OHD (2). Cortisol was low under baseline conditions in VBA− larvae, and the cortisol response to osmotic stress by VBA− larvae did not reach that of baseline levels in VBA+ larvae. This difference was also observed on a systemic level when we assessed the expression levels of glucocorticoid-responsive genes such as *pck1* and *fkbp5,* clearly indicating systemic glucocorticoid deficiency. However, overall Cyp21a2-deficient mutants showed some residual ability to synthesize cortisol and on a systemic level were less glucocorticoid deficient than our model of glucocorticoid deficiency caused by disruption of the mitochondrial redox cofactor to steroidogenesis ferredoxin 1b (fdx1b) (18).

This observation has several possible explanations. Fdx1b deficiency affects enzymatic steps in the synthesis to glucocorticoids: the catalytic activity of Cyp11a2 and Cyp11c1. Furthermore, as we collected 150 larvae for each of the steroid hormone measurements, we could not rule out that a proportion of heterozygous larvae might have been VBA−. However, we postulate the presence of another steroidogenic enzyme facilitating the residual 21-hydroxylation of the accumulating 17OHP in Cyp21a2-deficient larvae. The highly efficient interrenal 17-hydroxylase (Cyp17a2) (34) probably acts as an alternative 21-hydroxylating enzyme in 21-hydroxylase-deficient zebrafish larvae, because *cyp17a2* has the ability *in vitro* to convert 17OHP to 11-deoxycortisol (Fred Guengerich, personal communication). Thus, such a mechanism is the most likely explanation for measurable cortisol concentrations in Cyp21a2-deficient larvae.

Such a mechanism would also explain the residual cortisol synthesis leading to the less pronounced systemic glucocorticoid deficiency indicated by measurable expression levels of glucocorticoid-responsive genes *pck1* and *fkbp5*. Furthermore, the amounts of 21-deoxycortisol detected in our mutant larvae are likely to contribute as an additional factor, transactivating the expression of glucocorticoid-responsive genes, as has been demonstrated *in vitro* (5).

### Cyp21a2-deficient zebrafish larvae as a model of glucocorticoid deficiency

In contrast to mice, zebrafish generate cortisol as the main glucocorticoid with the same intermediates as humans, and as diurnal animals follow a similar circadian rhythm (13). Despite the fact that the murine deletion model of 21-hydroxylase has led to improved understanding of adrenal pathophysiology in CAH (8), it has been difficult to maintain homozygous murine *cyp21a2* deletion models (6, 8), making these an unsuitable tool to study systemic effects of 21OHD. Despite the lack of hyperandrogenism, our *in vivo* model of 21OHD resembles a number of key features of the human pathophysiology of 21OHD due to glucocorticoid deficiency. Thus, we believe that these mutant lines can be used to explore the whole-organism response to cortisol deficiency due to 21OHD. It appears that during the developmental stages studied, Cyp21a2-deficient zebrafish larvae do not synthesize excess androgens despite producing increased amounts of 17OHP. Thus, it appears that androgen precursors cannot enter the androgen biosynthesis pathway. This suggests that Cyp17a2 lacking 17,20 lyase catalytic function (34) is the predominant active interrenal enzyme during the studied timeframe and that Cyp17a1 might not be of functional relevance at 5 dpf. However, we believe that our *in vivo* model of 21OHD will be a useful tool to study the systemic consequences of cortisol deficiency modified by the systemic action of steroid hormone precursors such as 21-deoxycortisol.

## Conclusions

We have used a genetic engineering strategy to define a key step in glucocorticoid biosynthesis *in vivo*. By using molecular and biochemical approaches, we characterized the role of Cyp21a2 during zebrafish development and showed that it is necessary for interrenal cortisol synthesis. By using TALENs we have successfully generated *cyp21a2* mutant lines, disrupting the zebrafish steroid biosynthesis pathway, which was reflected in a steroid profile pathognomonic for 21OHD.

Our *cyp21a2* mutants will provide a valuable resource for exploring the impact of glucocorticoid deficiency and will provide insights into the regulation of other processes by steroid hormones, including development, behavior, and stress research.
