# Supplementary material for: Genetic Disruption of 21-Hydroxylase in Zebrafish Causes Interrenal Hyperplasia
Source: Endocrinology. 2017 Sep 13;158(12):4165–73. doi: 10.1210/en.2017-00549 (PMC5711382; doi:10.1210/en.2017-00549)
Supplement: Supplementary file 1 [file en.2017-00549.sf1.pdf]

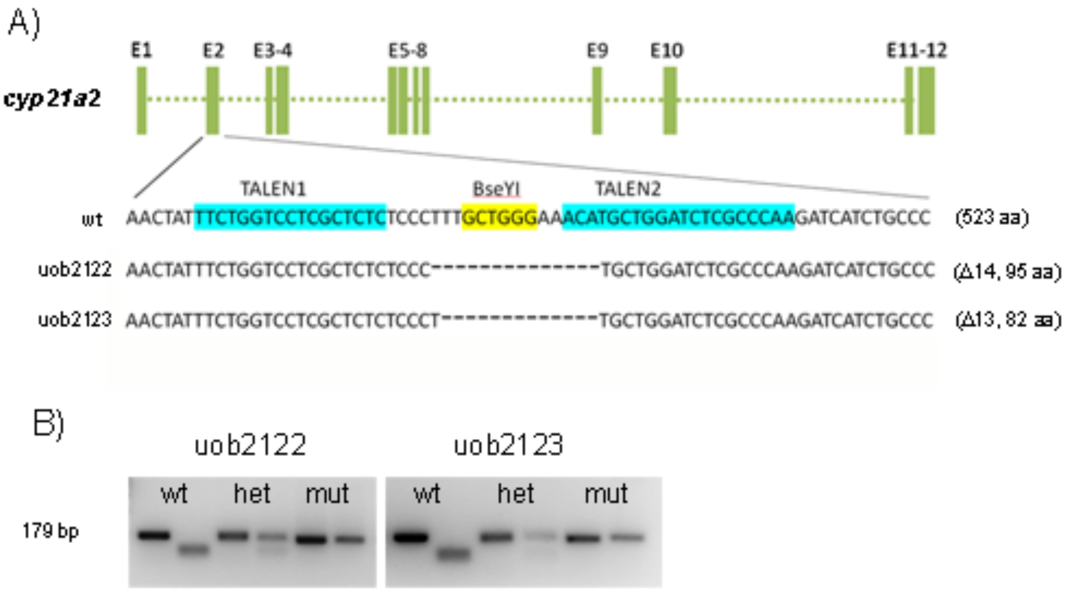

Supplementary Figure 1

A)

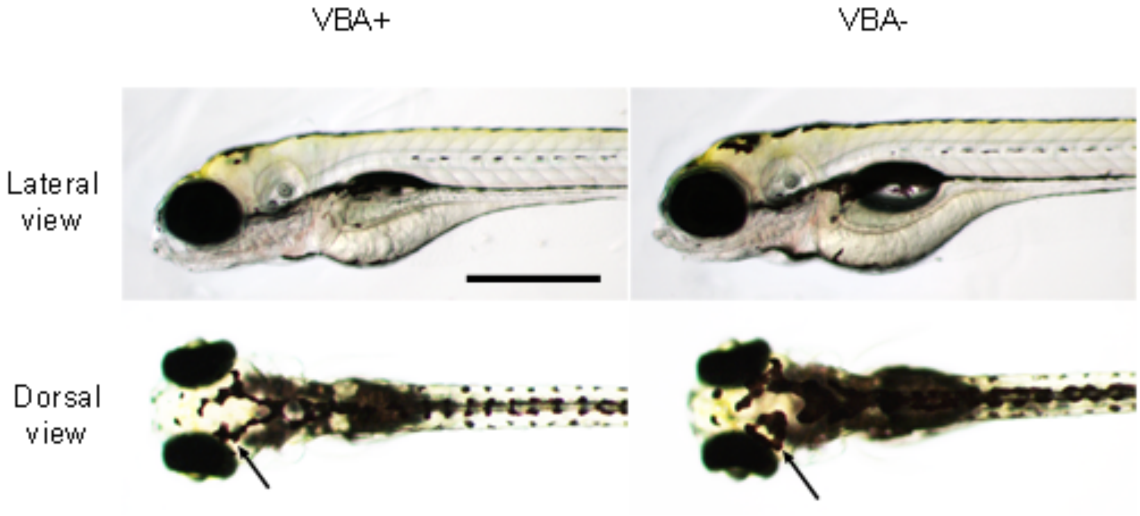

B)

Genotype phenotype correlation of Visual Background Adaption (VBA) phenotype			
**Line 2 (uob2122)**	*Genotype*		
Phenotype	Wild type	Heterozygous mutant	Homozygous mutant
VBA+ pale (n = 29)	4	**25**	0
VBA- dark (n = 48)	0	**5**	**43**
**Line 3 (uob2123)**	*Genotype*		
Phenotype	Wild type	Heterozygous mutant	Homozygous mutant
VBA+ pale (n = 9)	**4**	**5**	0
VBA- dark (n = 10)	0	0	**10**

Supplementary Figure 2

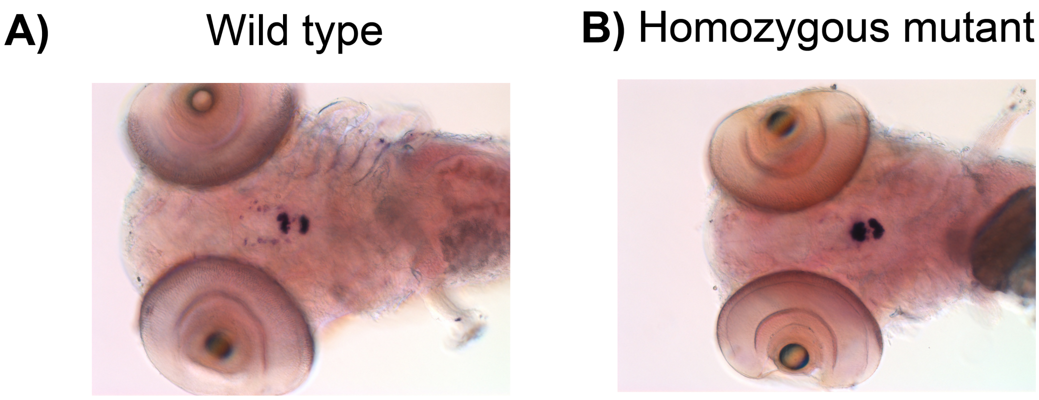

Supplementary Figure 3
