# Supplementary material for: Genetic Disruption of 21-Hydroxylase in Zebrafish Causes Interrenal Hyperplasia
Source: Endocrinology. 2017 Sep 13;158(12):4165–73. doi: 10.1210/en.2017-00549 (PMC5711382; doi:10.1210/en.2017-00549)
Supplement: Supplementary file 2 [file en.2017-00549.st1.pdf]

**Species**	**Identifier**	TILAPIA	**ZEBRAFISH**	CAVE_FISH	EEL	PUFFERFISH	PLATYFISH	MEDAKA	STICKLEBACK	GROUSE	CHICKEN	GOLDEN	RAT	MOUSE	GUINEA	COW	PIG	DOLPHIN	CAT	LYNX	DOG	WOLF	PANDA	RABBIT	HUMAN	GORILLA	CHIMPANZEE	ORANG_UTAN
TILAPIA	TILAPIA_tr|I3J3V0|	100	57.34	59.45	61.11	67.51	72.26	75.41	71.93	36.67	36.13	36.92	33.58	34.85	34.34	35	35.25	35.24	34.34	34.09	33.83	33.83	33.83	34.58	35.66	35.57	35.82	36.07
**ZEBRAFISH**	**ZEBRAFISH_tr|H0WEK6|**	**57.34**	**100**	**70.52**	**68.34**	**66.6**	**65.69**	**71.65**	**66.09**	**37.83**	**40.8**	**41.42**	**39.12**	**39.37**	**38.57**	**39.67**	**39.96**	**38.88**	**39.62**	**39.41**	**39.2**	**39.2**	**39.24**	**39.58**	**40.71**	**41.25**	**40.83**	**40.62**
CAVE_FISH	CAVE_FISH_tr|W5LAA4|	59.45	70.52	100	68.79	65.18	66.8	73.99	68.15	37.68	41.03	41.51	37.21	38.51	38.56	38.82	38.69	38.66	39.41	38.98	39.41	39.41	38.59	39.16	39.24	39.58	39.79	39.79
EEL	JAPANESE_EEL_tr|Q7ZZR9|	61.11	68.34	68.79	100	67.31	68.22	74.39	70.58	39.1	42.43	42.92	38.43	41.03	41.28	41.1	40.34	40.72	40.21	40	40.85	40.85	40.26	40.59	41.74	41.65	41.65	41.86
PUFFERFISH	SPOTTED_GREEN_PUFFERFISH_tr|H3DM74|	67.51	66.6	65.18	67.31	100	74.71	80.22	77.22	38.24	41.24	41.72	38.9	39.79	40.04	40.3	40.17	40.34	39.62	39.62	40.68	40.68	40.09	40.42	41.14	41.26	41.47	41.47
PLATYFISH	SOUTHERN_PLATYFISH_tr|M4AKU8|	72.26	65.69	66.8	68.22	74.71	100	84.3	77.95	38.57	40.43	41.13	38.46	40.43	39.61	40.09	39.74	40.13	38.97	38.76	39.19	39.19	39.66	39.57	40.51	40.43	40.85	40.64
MEDAKA	MEDAKA_tr|H2LRG4|	5.41	71.65	73.99	74.39	80.22	84.3	100	83.52	40.97	43.64	43.94	39.5	42.11	41.59	41.04	40.68	41.5	41.36	41.14	41.36	41.36	40.73	40.5	41.4	41.4	41.4	41.4
STICKLEBACK	STICKLEBACK_PTHR24281	71.93	66.09	68.15	70.58	77.22	77.95	83.52	100	38.15	40.94	41.2	38.56	40.51	40.98	40.59	40.25	39.58	39.7	39.49	39.7	39.7	39.53	40.72	41.23	41.14	41.35	40.93
GROUSE	BLACK_GROUSE_tr|I6PDD1|	36.67	37.83	37.68	39.1	38.24	38.57	40.97	38.15	100	88.29	90.4	38.7	40.19	41.53	41.9	41.67	43.74	43.44	42.96	43.2	43.2	43.68	40.76	41.09	40.76	41	41
CHICKEN	CHICKEN_tr|A5HUM5|	36.13	40.8	41.03	42.43	41.24	40.43	43.64	40.94	88.29	100	94.9	41.76	43.32	45.63	45.22	44.68	46.09	46.27	45.63	45.63	45.63	46.14	44.28	44.16	43.86	44.07	43.86
GOLDEN	GOLDEN_PHESANT_tr|I6YHU5|	36.92	41.42	41.51	42.92	41.72	41.13	43.94	41.2	90.4	94.9	100	42.61	44.42	46.97	46.55	46	47.64	47.4	46.75	46.75	46.75	47.49	45.16	45.26	44.95	45.16	44.95
RAT	RAT_sp|Q64562|	33.58	39.12	37.21	38.43	38.9	38.46	39.5	38.56	38.7	41.76	42.61	100	87.27	67.42	70.14	70.72	72.34	70.1	69.9	68.66	68.66	70.54	72.07	69.75	69.2	69.4	69.4
MOUSE	MOUSE_sp|P03940|	34.85	39.37	38.51	41.03	39.79	40.43	42.11	40.51	40.19	43.32	44.42	87.27	100	68.88	71.9	73.44	74.43	73.44	73.65	72.2	72.2	73.28	72.31	71.84	71.28	71.49	71.49
GUINEA	GUINEA _PIG	34.34	38.57	38.56	41.28	40.04	39.61	41.59	40.98	41.53	45.63	46.97	67.42	68.88	100	72.76	71.89	72.1	72.56	72.97	71.54	71.54	72.6	74.19	75.2	74.19	74.59	74.8
COW	COW_sp|P00191|	35	39.67	38.82	41.1	40.3	40.09	41.04	40.59	41.9	45.22	46.55	70.14	71.9	72.76	100	87.8	88.41	81.91	82.11	81.91	81.91	84.87	78.7	78.66	79.11	79.31	79.92
PIG	PIG_sp|P15540|	35.25	39.96	38.69	40.34	40.17	39.74	40.68	40.25	41.67	44.68	46	70.72	73.44	71.89	87.8	100	89	83.1	82.48	81.87	81.87	83.61	78.66	79.63	79.88	80.08	80.49
DOLPHIN	DOLPHIN_PTHR24281	35.24	38.88	38.66	40.72	40.34	40.13	41.5	39.58	43.74	46.09	47.64	72.34	74.43	72.1	88.41	89	100	83.1	82.48	82.48	82.48	84.63	78.14	79.51	79.15	79.55	79.96
CAT	CAT_sp|Q2LA60|	34.34	39.62	39.41	40.21	39.62	38.97	41.36	39.7	43.44	46.27	47.4	70.1	73.44	72.56	81.91	83.1	83.1	100	98.58	85.37	85.37	87.53	78.05	79.47	79.07	79.27	79.47
LYNX	LYNX_sp|Q2LA59|	34.09	39.41	38.98	40	39.62	38.76	41.14	39.49	42.96	45.63	46.75	69.9	73.65	72.97	82.11	82.48	82.48	98.58	100	86.18	86.18	87.93	78.66	79.47	79.07	79.27	79.27
DOG	DOG_sp|Q8WNW0|	33.83	39.2	39.41	40.85	40.68	39.19	41.36	39.7	43.2	45.63	46.75	68.66	72.2	71.54	81.91	81.87	82.48	85.37	86.18	100	100	89.16	77.64	78.25	77.85	77.85	77.85
WOLF	WOLF_sp|Q2LCM1|	33.83	39.2	39.41	40.85	40.68	39.19	41.36	39.7	43.2	45.63	46.75	68.66	72.2	71.54	81.91	81.87	82.48	85.37	86.18	100	100	89.16	77.64	78.25	77.85	77.85	77.85
PANDA	PANDA_tr|G1L6B8|	33.83	39.24	38.59	40.26	40.09	39.66	40.73	39.53	43.68	46.14	47.49	70.54	73.28	72.6	84.87	83.61	84.63	87.53	87.93	89.16	89.16	100	78.53	78.73	77.91	78.32	78.53
RABBIT	RABBIT_tr|G1SS94|	34.58	39.58	39.16	40.59	40.42	39.57	40.5	40.72	40.76	44.28	45.16	72.07	72.31	74.19	78.7	78.66	78.14	78.05	78.66	77.64	77.64	78.53	100	78.54	78.38	79.19	79.39
HUMAN	HUMAN_sp|P08686|	35.66	40.71	39.24	41.74	41.14	40.51	41.4	41.23	41.09	44.16	45.26	69.75	71.84	75.2	78.66	79.63	79.51	79.47	79.47	78.25	78.25	78.73	78.54	100	97.77	98.58	97.57
GORILLA	GORILLA_tr|G3RB24|	35.57	41.25	39.58	41.65	41.26	40.43	41.4	41.14	40.76	43.86	44.95	69.2	71.28	74.19	79.11	79.88	79.15	79.07	79.07	77.85	77.85	77.91	78.38	97.77	100	98.59	97.58
CHIMPANZEE	CHIMPANZEE_tr|H2RI19|	35.82	40.83	39.79	41.65	41.47	40.85	41.4	41.35	41	44.07	45.16	69.4	71.49	74.59	79.31	80.08	79.55	79.27	79.27	77.85	77.85	78.32	79.19	98.58	98.59	100	98.59
ORANG_UTAN	ORANG_UTAN_tr|H2PIM4|	36.07	40.62	39.79	41.86	41.47	40.64	41.4	40.93	41	43.86	44.95	69.4	71.49	74.8	79.92	80.49	79.96	79.47	79.27	77.85	77.85	78.53	79.39	97.57	97.58	98.59	100
